# Predicting survival of NSCLC patients treated with immune checkpoint inhibitors: Impact and timing of immune-related adverse events and prior tyrosine kinase inhibitor therapy

**DOI:** 10.3389/fonc.2023.1064169

**Published:** 2023-02-13

**Authors:** Michael R. Sayer, Isa Mambetsariev, Kun-Han Lu, Chi Wah Wong, Ashley Duche, Richard Beuttler, Jeremy Fricke, Rebecca Pharoan, Leonidas Arvanitis, Zahra Eftekhari, Arya Amini, Marianna Koczywas, Erminia Massarelli, Moom Rahman Roosan, Ravi Salgia

**Affiliations:** ^1^ Department of Pharmacy Practice, Chapman University School of Pharmacy, Irvine, CA, United States; ^2^ Department of Medical Oncology and Therapeutics Research, City of Hope National Medical Center, Duarte, CA, United States; ^3^ Department of Applied AI and Data Science, City of Hope National Medical Center, Duarte, CA, United States; ^4^ Department of Pathology, City of Hope National Medical Center, Duarte, CA, United States; ^5^ Department of Radiation Oncology, City of Hope National Medical Center, Duarte, CA, United States

**Keywords:** non-small cell lung cancer, immunotherapy, immune-related adverse events, tyrosine kinase inhibitors (TKI), machine learning, survival analysis

## Abstract

**Introduction:**

Immune checkpoint inhibitors (ICIs) produce a broad spectrum of immune-related adverse events (irAEs) affecting various organ systems. While ICIs are established as a therapeutic option in non-small cell lung cancer (NSCLC) treatment, most patients receiving ICI relapse. Additionally, the role of ICIs on survival in patients receiving prior targeted tyrosine kinase inhibitor (TKI) therapy has not been well-defined.

**Objective:**

To investigate the impact of irAEs, the relative time of occurrence, and prior TKI therapy to predict clinical outcomes in NSCLC patients treated with ICIs.

**Methods:**

A single center retrospective cohort study identified 354 adult patients with NSCLC receiving ICI therapy between 2014 and 2018. Survival analysis utilized overall survival (OS) and real-world progression free survival (rwPFS) outcomes. Model performance matrices for predicting 1-year OS and 6-month rwPFS using linear regression baseline, optimal, and machine learning modeling approaches.

**Results:**

Patients experiencing an irAE were found to have a significantly longer OS and rwPFS compared to patients who did not (median OS 25.1 vs. 11.1 months; hazard ratio [HR] 0.51, confidence interval [CI] 0.39- 0.68, P-value <0.001, median rwPFS 5.7 months vs. 2.3; HR 0.52, CI 0.41- 0.66, P-value <0.001, respectively). Patients who received TKI therapy before initiation of ICI experienced significantly shorter OS than patients without prior TKI therapy (median OS 7.6 months vs. 18.5 months; P-value < 0.01). After adjusting for other variables, irAEs and prior TKI therapy significantly impacted OS and rwPFS. Lastly, the performances of models implementing logistic regression and machine learning approaches were comparable in predicting 1-year OS and 6-month rwPFS.

**Conclusion:**

The occurrence of irAEs, the timing of the events, and prior TKI therapy were significant predictors of survival in NSCLC patients on ICI therapy. Therefore, our study supports future prospective studies to investigate the impact of irAEs, and sequence of therapy on the survival of NSCLC patients taking ICIs.

## 1 Introduction

Tyrosine kinase inhibitors (TKIs) have shown an improvement in overall survival (OS) and progression-free survival (PFS) in non-small cell lung cancer (NSCLC) (1, 2). Despite the advancement, disease progression remains inevitable for advanced metastatic NSCLC cases. Immunotherapy is a standard of care for these patients without a druggable mutation using immune checkpoint inhibitors (ICIs). ICI-based therapies can be used alone or in combination with platinum-based chemotherapy (1). Although programmed death-ligand 1 (PD-L1) status using immunohistochemistry assays has been used widely as a biomarker for ICI therapy, only 20-40% of the PD-L1 positive patients benefit from ICIs (3). Additionally, some patients receiving ICIs develop immune-related adverse events (irAEs) (4). ICI-based therapies target immune checkpoint proteins found on immune regulatory T cells that interact with antigens on tumor cells or antigen-presenting cells (5). Two classes of inhibitors are used to target the negative immunomodulatory proteins, programmed death-1 (PD-1) and its respective ligand, PD-L1. Specifically for metastatic NSCLC, PD-1 inhibitors, including pembrolizumab, nivolumab, cemiplimab, and PD-L1 inhibitor atezolizumab can be used alone or in combination with platinum-based chemotherapy. In both cases, survival time has been shown to significantly increase in NSCLC patients compared to patients receiving chemotherapy alone (6).

Despite the ICI effectiveness, the onset of irAEs can occur late after the initiation of treatment compared to chemotherapy (7). irAEs result from dysregulation in normal immune self-tolerance affecting multiple organ systems, most commonly including the endocrine system, gastrointestinal tract, liver, and skin (8). The median time to irAE onset ranges from 4.9 weeks for gastrointestinal irAEs to 30.3 weeks for pulmonary irAEs during ICI therapy (9, 10). Although irAEs are closely related to ICIs, clinical trials paying particular attention to irAEs have been limited. ICI treatments are typically discontinued if a serious irAE occurs. Thus, the need for improved predictive biomarkers for NSCLC has led to the recent investigation of irAEs and their effect on patient outcomes. Preliminary evidence does support an association between irAEs and improved therapy outcomes in NSCLC patients taking ICIs, except in patients experiencing more severe events (11–13). Studies also report that early irAEs are associated with better outcomes after immunotherapy (14–17). Specific systemic effects, such as pneumonitis, thyroid dysregulation, and multi-system irAEs, have been investigated for their links to therapy outcomes (18–21). However, we still lack consensus on whether irAEs, their timing, or specific types are indicative of positive therapeutic outcomes.

The role of ICI therapy in patients with a history of TKI therapy is unknown. Preliminary evidence shows reduced survival time on ICI monotherapy in NSCLC patients with known oncogenic drivers compared to TKI and chemotherapies (22). While comprehensive evaluation of the efficacy of ICI in NSCLC post-TKI treatment resistance was not readily available in the literature, a few have investigated outcomes in patients receiving EGFR-targeted TKI therapy. Initial evidence suggests that EGFR mutation-positive patients taking ICI inhibitor therapy after TKI therapy have worsened survival times compared to those that took it before (23, 24). Such associations need to be further explored for other TKI therapies compared to the timing of ICI therapy.

Survival analysis in NSCLC patients is challenging due to the heterogeneity of factors influencing therapeutic outcomes (25). Non-conventional response pattern related to ICI makes it even more challenging to predict survival (26). In addition to classical survival analysis techniques, such as Kaplan Meier (KM) survival curves and Cox-proportional hazards (COXPH) models assessing mortality risk, models predicting survival of NSCLC patients, including logistic regression and machine learning (ML) approaches (e.g., penalized logistic regression, support vector machines, random forests, etc.) can provide insight into complex interaction among various clinical and molecular characteristics. Thus, ML has the potential to address these limitations and has been implemented in NSCLC patients predicting survival utilizing data related to gene expression and DNA methylation data (27, 28). Although ML approaches have been used to predict the occurrence of irAEs in NSCLC patients (29, 30), we are not aware of studies that have used irAE to predict clinical outcomes using ML.

Here, we conducted a single-center retrospective study to investigate irAE occurrence, the timing of irAEs, and the influence of prior TKI therapy on clinical outcomes in NSCLC patients receiving ICIs. We compared the predictive performance of models assessing survival using statistical and ML approaches.

## 2 Methods

### 2.1 Participants

A retrospective analysis was performed on 423 City of Hope cancer patients and de-identified clinical data was obtained from the electronic health records and Thoracic Oncology Registry (THOR) according to the guidelines of the institutional review board-approved protocol. Specifically, treatment history, sex, smoking status, tumor stages, age at diagnosis, vital status, tissue and molecular testing results, therapeutic agents, and progression during the follow-up were abstracted from the electronic health record (EHR). Clinical histological diagnoses of lung cancer were retrieved from the EHR pathology reports, which were confirmed through hematoxylin-eosin and immunostained slides by a board certified pathologist.

### 2.2 Eligibility criteria

Inclusion criteria included patients 1) diagnosed with NSCLC, 2) treated with an ICI, 3) received the first dose of ICI between 2014 to 2018, and 4) 18 years of age or older. The follow-up period for patients lasted through December 31, 2020. Patients receiving ICIs as monotherapy, in combination with chemotherapy, and receiving multiple ICI agents in combination were considered. Exclusion criteria include patients with Fluorescence *in situ* Hybridization data available without molecular test data, tumor sample quantity insufficient for molecular characterization, and TKI therapies unsupported by available molecular test results. Only the earliest molecular testing reports were included in the study in cases of multiple molecular test reports. A total of 423 patients were evaluated and 354 were identified as meeting the criteria outlined (**Supplemental Figure 1**). The study was approved by the City of Hope (COH) and Chapman University Institutional Review Boards (IRB) under IRB #18343 and IRB-21-193, respectively.

### 2.3 Clinical outcome measures

Primary outcomes were OS and real-world progression-free survival (rwPFS). OS was calculated from the start date of an ICI to the date of death or last recorded visit according to the medical records. Dates of rwPFS were retrospectively evaluated in the physician notes documenting progression from the EHR following a previously described validation framework (31). Additionally, 1-year OS and 6-month rwPFS endpoints were used for predictive modeling.

### 2.4 Measures of immune-related adverse events

irAEs were defined as adverse events occurring with immune-mediated etiology that potentially require immune-modulating or endocrine therapy. Whether these irAEs occurred, the number of days after ICI initiation they occurred, categorized based on how they impacted the body and PD-L1 staining status, characterized as <1%, 1-49%, and ≥50%, were collected (32, 33). Early irAEs were defined as irAEs that occurred within 69 days, median days to irAE in the cohort after commencing ICI therapy. irAEs were additionally categorized based on the organ systems affected due to the ICI treatment, such as endocrine, dermal, gastrointestinal, and respiratory. Other clinical data and molecular testing data were also acquired.

### 2.5 Analytical methods

Descriptive statistics were used for all demographic variables. The frequencies of *EGFR, KRAS, ALK, ROS1, BRAF, MET, RET, NTRK1/2/3, TP53, CDKN2A, CDKN2B, LRP1B, STK11, PIK3CA, SMARCA4, ERBB2*, and *ARID1A* genes were analyzed. Genes were selected for analysis based on National Comprehensive Cancer Network (NCCN) guidelines for NSCLC management related to targeted therapies (34). Other genes were selected due to their high prevalence in the patient cohort. A variant not classified as the variant of unknown significance was considered a positive somatic mutation for each gene analyzed. KM survival analyses with log-rank statistical tests were used to assess OS and rwPFS. COXPH methods were implemented to determine hazard ratios relevant to outcome variables. Multivariate COXPH models were created with OS and rwPFS endpoints at 1-year and 6-months, respectively. Overall statistical significance of model fit was assessed with a log-rank statistical test, with the significance of individual covariates being assessed based on Wald statistics and their associated P-values.

### 2.6 Predictive modeling

Using statistical and ML approaches, we built two predictive models for classifying patients: 1) OS at 1-year, and 2) rwPFS at 6-months. Training and test datasets were created from the collected data with a randomized 80:20 split. Chi-square test of independence was applied to investigate the association among categorical variables in the training dataset. The association within 18 variables representing patient demographics and treatment data was visualized using a heatmap of p-values resulting from the pairwise Chi-square tests (**Supplemental Figure 2**; **Supplement Table 1**). Area Under Receiver Operating Characteristics (AUROC), Area Under Precision-Recall Curve (AUPRC), accuracy, and F1 score were used to assess model performance. In addition, the holdout testing set was further stratified into two distinct prognostic subgroups (i.e., < median survival score, >= median survival score) based on the predicted survival scores. KM method and log-rank tests of OS and rwPFS were performed on these two groups.

#### 2.6.1 Statistical modeling

As a traditional statistical modeling approach, logistic regression (LR) modeling was implemented to predict 1-year OS and 6-month rwPFS outcomes. Two models were created to predict each outcome (1) Baseline LR (BLR) models utilizing all potential predictive variables in the dataset, and (2) optimized LR (OLR) models based on an alkaline information criterion (AIC) minimization strategy. An exhaustive algorithm created OLR models with the training data attempting every potential combination of the predictor variables, ultimately selecting the model with the lowest AIC. LR modeling was performed in R-Studio version 4.1.1 with stats (v 3.6.2), bestglm (v 0.37.3), and caret (v 3.45) packages.

#### 2.6.2 Machine learning modeling

A logistic regression with elastic-net regularization for classification was used as an ML approach. A 10-fold cross-validation was used to identify optimal model hyperparameters within the training set. The optimized model was then applied to the holdout test set and the predictions were used as survival scores. To facilitate the interpretation of the machine learning models, we applied the SHapley Additive exPlanations (SHAP) method to compute explanations for each individual prediction based on the associated SHAP values for each feature (35). The overall feature importance was obtained by calculating the mean absolute SHAP values of individual features. Python 3.9.7, scikit-learn (v 1.0.2), lifelines (v 0.26.4), and shap (v 0.39.0) were used for ML classification and explanation.

## 3 Results

Of the 354 patients included in the study, 54% were male, 57% were White, 80% were Stage IV and 77% were Adenocarcinoma NSCLC patients (**Table 1**). A total of 53 patients received chemotherapy in combination with immunotherapy, 286 patients received immunotherapy alone, and 15 patients received a combination immunotherapy, such as nivolumab and ipilimumab combination. 72% of patients received anti-PD-1 agents while others received anti-PD-L1 agents and 18% of patients received oncogene targeted TKIs. Overall, 60.45% of patients had a positive mutation at least in one gene analyzed. While everyone receiving a TKI had a mutation, 52.23% of patients who did not receive a prior TKI had a mutation, the most frequently mutated ones are in *TP53*, *KRAS*, and *CDKN2A* genes (**Supplemental Table 2**; **Supplemental Figures 3, 4**).

**Table 1:** Characteristics of patient population identified from the retrospective chart review.

Characteristics of the Study Population (N= 354)	Category	Statistics (% or IQR)
**Overall survival (OS) status, n (%)**	Alive Deceased	147 (41) 207 (59)
**1-Year OS, n (%)**	Alive Deceased	173 (49) 181 (51)
**Real-world progression free survival (rwPFS), n (%)**	No Progression Progression	58 (16) 296 (84)
**6-month rwPFS, n (%)**	No Progression Progression	153 (43) 201 (57)
**OS in days**	Median	343
	IQR	109 - 661
**rwPFS in days**	Median	87
	IQR	49- 253
**Sex, n (%)**	Male	190 (54)
	Female	164 (46)
**Histology, n (%)**	Adenocarcinoma	271 (77)
	Squamous Cell Carcinoma	72 (20)
	Other or Unknown	11 (3)
**Race, n (%)**	White	203 (57)
	Asian	69 (19)
	Black	13 (4)
	Other or Unknown	67 (19)
**Stage, n (%)**	I or II	16 (4)
	III	40 (11)
	IV	283 (80)
**Smoking status, n (%)**	Current/Former Smoking	246 (70)
	No Smoking	108 (30)
**Class of immunotherapy, n (%)**	Anti-PD-1	254 (72)
	Anti-PD-L1	100 (28)
**Immunotherapy combination status, n (%)**	Monotherapy	286 (81)
	With Chemotherapy	53 (15)
	Combination ICI Therapy	15 (4)
**TKI Therapy** **Target and Timing, n (%)**	Anti-EGFR TKI After ICI	6 (1.7)
	Anti-ALK TKI After ICI	2 (0.6)
	Anti-BRAF TKI After ICI	1 (0.3)
	Anti-MET TKI After ICI	1 (0.3)
	Anti-EGFR TKI Prior to ICI	42 (12)
	Anti-ALK TKI Prior to ICI	8 (2.2)
	Anti-BRAF TKI Prior to ICI	2 (0.6)
	Anti-MET TKI Prior to ICI	0 (0)
	Clinical Trial TKI	1 (0.3)
**Programmed death-ligand 1 (PD-L1) Range, n (%)**	PD-L1 Range ≥50%	94 (27)
	PD-L1 Range 1-49%	56 (16)
	PD-L1 Test Negative	76 (21)
**Immune-related adverse events**	Yes	152 (43)
**(irAE) occurrence, n (%)**	No	202 (57)
**irAE timing (early <=69 days and**	Early irAE	76 (21)
**late > 69 days), n (%)**	Late irAE	76 (21)
**Type of irAE, n (%)**	Categorized irAE	76 (21)
	General irAE	76 (21)

Patient demographics data are represented as count data with the percentage of the total patient population represented given in parenthesis. Overall survival and real-world progression free days are described with median and interquartile range (IQR).

### 3.1 The impact of irAEs and TKI therapy on survival

**Figure 1:**
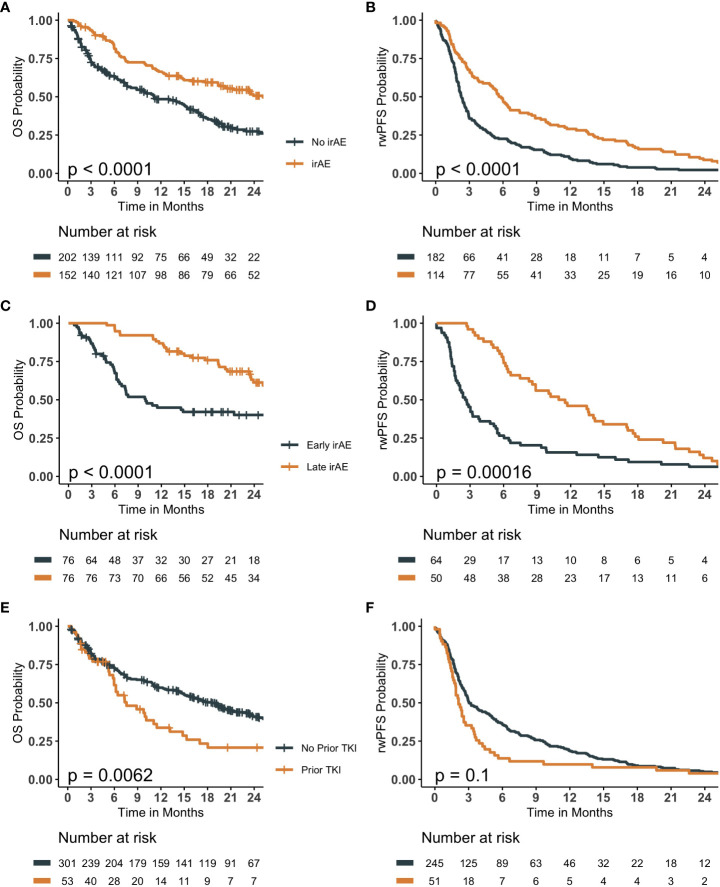
Kaplan Meier survival curves evaluating the role of immune-related adverse events (irAE) and history of tyrosine kinase inhibitor (TKI) therapy prior to immune checkpoint inhibitor (ICI) therapy on overall survival (OS) and real-world progression free survival (rwPFS). **(A, B)** compare patients experiencing an irAE to those that did not, evaluating OS and rwPFS, respectively. **(C, D)** compare patients experiencing an early-irAE to those experiencing a late-irAE event, evaluating OS and rwPFS, respectively. **(E, F)** compares patients who received a TKI prior to ICI therapy to those that did not, evaluating OS and rwPFS, respectively. All p-values are the result of a log-rank statistical test comparing the survival probability of the groups.

The median follow-up time was 11.45 months (IQR 3.6 - 22.0 months), of which 43% of the patients experience irAEs. The occurrence of an irAE, the timing of the irAE and the role of prior TKI were evaluated for OS and rwPFS (**Figure 1**). The occurrence of an irAE was associated with longer OS (median 25.1 vs 11.1 months, hazard ratio [HR] 0.51, confidence interval [CI] 0.39- 0.68, P-value <0.001) and rwPFS (median 5.7 vs 2.3 months, HR= 0.52, CI: 0.41- 0.66, P- Value <0.001). Significantly longer OS and rwPFS in patients experiencing later irAE were observed compared to early events (HR= 0.38, CI: 0.24- 0.6, P-value < 0.001, HR= 0.49, CI: 0.33 -0.71, P-value< 0.001, respectively). Additionally, patients receiving TKI prior to ICI therapy had shorter OS (7.6 months vs 18.5 months, HR 1.6, CI 1.1-2.3, P-value<0.01). Although not statistically significant, a similar pattern was observed for rwPFS (2.1 months vs 3 months, HR 1.3, CI 0.95-1.8, P-value= 0.1). The ten patients in the cohort receiving TKIs after ICI therapy showed significantly improved overall survival compared to those receiving them prior to ICI therapy (median 24.6 vs 7.57 months, HR= 1.64, CI: 1.15-2.35, P-value = 0.04; **Supplemental Figure 5A**). A similar trend was observed with rwPFS, however, it was not statistically significant (**Supplemental Figure 5B**; **Supplemental Table 3**). After adjusting for other variables, age at diagnosis, stage IV, prior TKI therapy, general irAE, categorized irAE, early irAE and PD-L1 range 50-100% were statistically significant predictors of OS (**Supplemental Figure 6**), while general irAE, categorized irAE, and early irAE were statistically significant predictors of rwPFS (**Supplemental Figure 7**).

### 3.2 Predictive modeling

**Figure 2:**
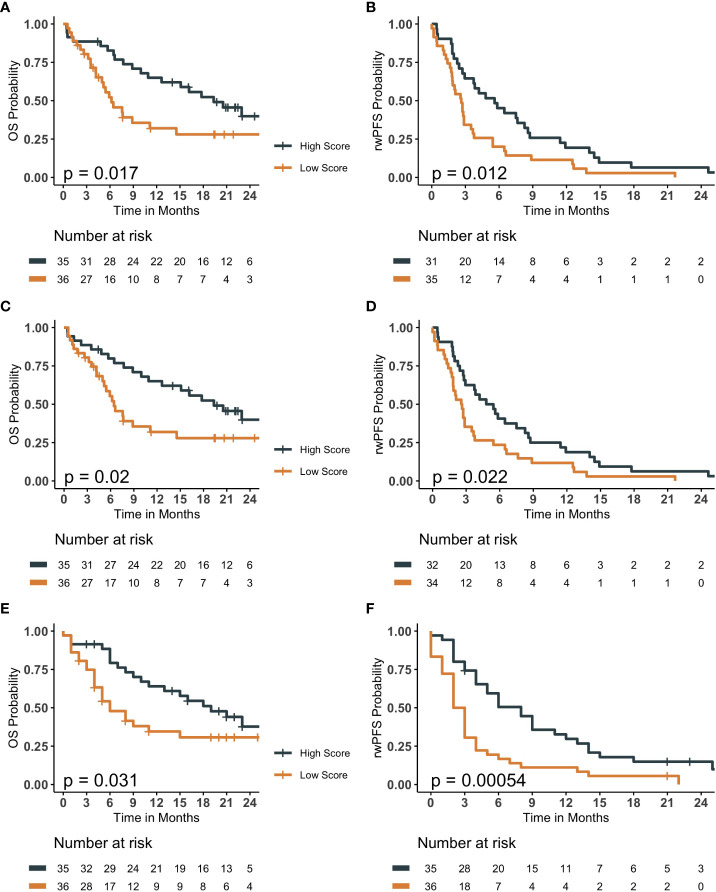
Kaplan Meier survival curves comparing patients assigned high survival scores compared to low survival scores for baseline and optimized logistic regression models in addition to elastic-net logistic regression model for overall survival (OS) and real-world progression free survival (rwPFS). Prediction results of baseline logistic regression models **(A, B)**, optimized logistic regression models **(C, D)**, and elastic-net logistic regression model **(E, F)** with Kaplan Meier survival plots. **(A, C, E)** are OS, with **(B, D)**, and F being rwPFS. Patients with survival scores higher than the median cut-off had significantly longer OS and rwPFS than those with lower survival scores. P-values less than 0.05 indicates a statistically significant probability from the log-rank statistical test that the high and low score groups have different survival probability.

The accuracy and the F-1 scores of 1-year OS models were the highest for the OLR model, while AUROC and AUPRC were the highest for the ML model (**Table 2**; **Supplemental Table 4**, **Supplemental Figure 8**). The median survival scores by BLR, OLR and ML models for 1-year OS was 0.483 (IQR 0.36- 0.58), 0.473 (IQR 0.38-0.60), and 0.46 (IQR 0.38 - 0.58), respectively. Patients with high survival scores had significantly longer median OS (BLR 19.3 vs 6.2 months; P-value= 0.017; OLR 19.33 vs 6.43 months; P-value= 0.02; ML 16 vs. 5 months; P-value <0.05) compared to those with lower scores (**Figure 2**). On the other hand, the performance metrics were similar for all 6-months rwPFS models, OLR model performing the worst for all metrics (**Table 2**; **Supplemental Table 4**). The median survival scores from BLR, OLR and ML models for 6-month rwPFS was 0.487 (IQR= 0.353- 0.603), 0.450 (IQR= 0.357- 0.559), and 0.52 (IQR: 0.39 to 0.65), respectively. Patients with higher survival scores had significantly longer rwPFS compared those with lower scores for all models (BLR 5.6 vs 2.63 months, P-value= 0.012; OLR 5.12 vs 2.60 months, P-value= 0.022; ML 6 vs. 2 months, P-value <0.001; **Figure 2**).

**Table 2:** Evaluation of model performance.

	1-Year OS Survival Baseline Logistic Regression Model	1-Year OS AIC Optimized Logistic Regression Model	1-Year OS Machine Learning Model	6-Months rwPFS Baseline Logistic Regression Model	6-Months rwPFS AIC Optimized Logistic Regression Model	6-Months rwPFS Machine Learning Model
*Accuracy*	0.70	0.75	0.70	0.63	0.57	0.63
*AUROC*	0.76	0.78	0.8	0.64	0.63	0.65
*AUPRC*	0.68	0.69	0.74	0.44	0.42	0.44
*F1-Score*	0.67	0.71	0.66	0.56	0.42	0.59

Metrices used to evaluate performance of various models implemented are accuracy, Area Under the Receiver Operator Curve (AUROC), Area Under Precision-Recall Curve (AUPRC) and F-1 score. All measures range between 0-1.

OS, Overall survival; rwPFS, real-world progression-free survival; AIC, Akaike information criterion.

**Figure 3:**
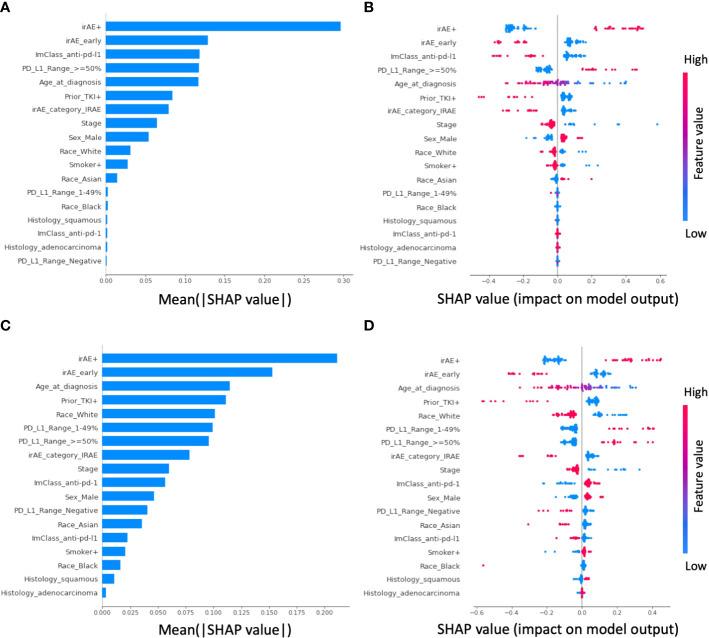
Shapley additive explanations (SHAP) analysis of feature importance in the elastic-net logistic regression-based prediction. For prediction of overall survival (OS) at 1 year, **(A)** shows the feature importance ranking from high (top) to low (bottom) based on the mean absolute SHAP values, whereas **(B)** shows the distribution and the impact of each feature on the model output. In the beeswarm plot, each row corresponds to one feature and each dot represents one patient’s data. The colors represent the feature value (red and blue for larger and smaller values, respectively). A positive SHAP value indicates the likelihood of having OS greater than 1 year increased, whereas a negative SHAP value indicate otherwise. For example, patients with irAEs (i.e., irAE+) have increased likelihood of OS greater than 1 year. For prediction of real-world progression free survival (rwPFS), the corresponding feature importance plot and the beeswarm plot is shown in panel **(C, D)**, respectively.

For the 1-year OS ML model, the five most important features were patients with irAE, early irAE event, anti-PDL1 treatment, PD-L1 expression level greater or equal to 50%, and age at diagnosis (**Figures 3A, B**). Patients with irAE or PD-L1 expression levels greater or equal to 50% have an increased probability of OS at 1-year, whereas patients with early irAE events, anti-PDL1 treatment, or older age at diagnosis have decreased probability of OS at 1-year. Similarly, for the 6-month rwPFS model, the five most important features were patients with irAE, early irAE event, age at diagnosis, patients who had received TKI prior to an ICI, and race (White) (**Figures 3C, D**). Patients with irAE have an increased probability of rwPFS at 6-months. In contrast, patients with early irAE events, older age at diagnosis, TKI prior to the immunotherapy, or race being White have decreased probability of rwPFS at 6-months.

## 4 Discussion

We evaluated the impact of irAEs and prior TKI therapy on OS and rwPFS in NSCLC patients receiving ICI therapy. 354 COH NSCLC patients were eligible for this retrospective cohort study. In addition, 6-month rwPFS and 1-year OS were predicted with LR and ML techniques. Regardless of the approach used, irAE occurrence and the timing of irAE were predictive of survival in patients receiving ICIs even after adjusting for other clinical factors. Our study identified a strong association between the occurrence of irAEs and improved OS and rwPFS in patients experiencing irAEs as compared to those that do not. Predictive modeling with LR and ML approaches further confirmed this relationship, with variables related to irAEs being strongly significant and associated with an improved likelihood of achieving 1-year OS and 6-month rwPFS endpoints.

Recent studies have highlighted the relationship between irAEs, their timing, and their severity with OS and rwPFS outcomes comparable to our findings. In a study of 152 NSCLC patients receiving ICI therapy, patients experiencing later irAEs (defined as after 3 months of ICI therapy initiation) had significantly longer OS and PFS than those with early irAEs and no irAE (median 30.9 months, 14.2 months, and 9.1 months, respectively) (18). In another study, patients experiencing irAEs had significantly higher OS and PFS (13). Similar results were also observed in another larger study of 559 NSCLC patients taking ICIs (16). Additional sub-analysis showed patients experiencing dermal irAEs, endocrine irAEs, and less severe irAEs had significantly higher OS and PFS than otherwise classified events (16). Rechallenging patients with ICI therapy after discontinuation due to irAEs resulted in improved OS compared to those without (36). Additionally, trials such as CheckMate 9LA evaluating ICI with chemotherapy combination therapy have shown that patients that discontinue ICI or dose reduction owing to irAEs still experience similar therapeutic benefits to those that do not (37).

In cases with resistance to TKIs targeting EGFR-specific oncogenes, strategies with combinations of targeted TKI therapies have been utilized with limited success (38, 39). Given the impact of ICIs on improving survival, exploring whether they can improve outcomes in TKI-resistant patients is a logical step. However, our results show reduced survival time in patients receiving ICI therapy after TKI therapy compared to patients that do not. Recent evidence from trials suggests ICIs offer no therapeutic benefit to EGFR mutant NSCLC patients with respect to overall survival time (37, 40–42). Several studies investigating the activity of immunotherapy, specifically PD-1 blockade in EGFR mutant NSCLC, reported poor response and efficacy (43–45). Furthermore, combination therapy approaches of ICI and targeted therapy have resulted in increased grade 3 or higher toxicities with no improvement in response or survival outcomes (46–48).

Here, we demonstrated that ML would allow providers to gain similar insights into the data as more established approaches. These efforts are important, as studies showed that providers could be reluctant to embrace artificial intelligence and ML approaches (49–51). Compared to traditional multivariate logistic regression, we show that ML models such as the elastic-net logistic regression used in this study could achieve improved or similar performance predicting survival. While ML approaches often achieve superior predictive performance compared to traditional statistical methods, they are commonly thought of as a “black box” predictor without explicit explanation, limiting their clinical utility (52). To overcome this issue, we applied the SHAP method to explain ML prediction by computing the contribution of each clinical feature to the prediction. For each patient, the model’s risk prediction can be decomposed into a set of SHAP values associated with the importance of clinical features, allowing clinicians and researchers to interpret and visualize the decision-making process made by the algorithm.

There are several limitations of this study. This was a single-center retrospective study using relatively few predictive variables. Subsequent studies could include additional variables relevant to irAEs, such as the irAE severity, role of immunosuppressants limiting the irAE impact in predicting outcomes (53). The role of genomics needs further exploration since preliminary evidence suggests certain mutations are biomarkers, such as *EGFR*, *MET*, *KRAS*, and *TP53*, influencing response to ICI therapy (54–57). Additionally, mutation data came from heterogeneous tests and sources of tumor samples (i.e., tissue or blood). We considered identified mutations from tissue and liquid biopsies in supporting TKI therapies regardless of the technology used. Despite the high concordance between tissue and blood biopsies, there is still variation in mutation detection (58). Most of our patients in the cohort receiving TKIs received EGFR-and ALK-targeted therapy. Therefore, our findings of the sequence of TKI and ICI need to be further investigated in NSCLC patients with diverse targetable mutations. While our study size was relatively large compared to prior studies investigating irAEs in NSCLC patients, the patient population had limited representation for stages of cancer, ethnicity, histology, and other features. The patient population was primarily advanced NSCLC patients who had failed prior therapies before initiating ICIs. Therefore, future larger multi-institutional prospective studies with a single molecular test are a must to understand the relationship between irAEs and their timing with ICI therapy outcomes.

## 5 Conclusion

irAEs and their timings were significantly associated with the OS and rwPFS, with patients experiencing these events later in their course of therapy having higher survival probabilities and more likely to achieve 1-year OS and 6-month rwPFS endpoints. The impact of TKI therapy prior to ICI therapy was also strongly associated with reduced OS and rwPFS times. irAE occurrences, timing, and prior TKI therapy were important factors in predicting 1-year OS. Thus, our results support future prospective studies to investigate these associations further.

## Data availability statement

The data and the analyses codes are available at https://github.com/sayer108/NSCLCStudy.

## Ethics statement

The studies involving human participants were reviewed and approved by City of Hope Institutional Review Board (IRB#18343). Patient informed consent (45 CFR 46.116) and HIPAA authorization (45 CFR 164.512) were waived in accordance with City of Hope Institutional Review Board approval under Federal Policy for the Protection of Human Subjects.

## Author contributions

Concept and design: MS, K-HL, MR, IM, RS. Acquisition, analysis, or interpretation of the data: all authors. Drafting of the manuscript: MS, K-HL, CW, IM, MR, RS. Critical revision of the manuscript: all authors. Statistical analysis: MS, K-HL, CW, MR. Supervision: MR, IM, RS. All authors contributed to the article and approved the submitted version.
