# Supplementary material for: Predicting survival of NSCLC patients treated with immune checkpoint inhibitors: Impact and timing of immune-related adverse events and prior tyrosine kinase inhibitor therapy
Source: Front Oncol. 2023 Feb 13;13:1064169. doi: 10.3389/fonc.2023.1064169 (PMC9968834; doi:10.3389/fonc.2023.1064169)
Supplement: Supplementary file 1 [file DataSheet_1.docx]

**Supplemental Table 1: Predictive variables utilized predicting 1-year overall survival (OS) and 6-month real-world progression-free survival (rwPFS) outcomes**

Count data in the training and test data set columns shows number of patients within each category for both data sets.

| **Characteristic** | **Testing Data Set** | **Training Data Set** | **Characteristic** | **Testing Data Set** | **Training Data Set** |
| --- | --- | --- | --- | --- | --- |
| \| **Overall survival (OS) status at 1 year** \| \| --- \| \| Alive \| \| Deceased \| | \|  \| \| --- \| \| 143 \| \| 140 \| | \|  \| \| --- \| \| 30 \| \| 41 \| | \| **Class of Immunotherapy** \| \| --- \| \| Anti-PD-L1 \| \| Anti-PD-1 \| | \|  \| \| --- \| \| 79 \| \| 204 \| | \|  \| \| --- \| \| 19 \| \| 52 \| |
| \| **Real-world progression free survival (rwPFS) status at 6 months** \| \| --- \| \| No Progression \| \| Progression \| | \|  \| \| --- \| \| 127 \| \| 156 \| | \|  \| \| --- \| \| 26 \| \| 45 \| | \| **TKI Therapy Received** \| \| --- \| \| Received TKI \| \| No TKI Therapy \| | \|  \| \| --- \| \| 52 \| \| 231 \| | \|  \| \| --- \| \| 11 \| \| 60 \| |
| \| **Sex** \| \| --- \| \| Male \| \| Female \| | \|  \| \| --- \| \| 151 \| \| 132 \| | \|  \| \| --- \| \| 39 \| \| 32 \| | \| **TKI Therapy Timing** \| \| --- \| \| Prior to ICI \| \| After ICI \| | \|  \| \| --- \| \| 43 \| \| 9 \| | \|  \| \| --- \| \| 10 \| \| 1 \| |
| \| **Histology** \| \| --- \| \| Adenocarcinoma \| \| Squamous Cell \| \| Other \| | \|  \| \| --- \| \| 215 \| \| 58 \| \| 10 \| | \|  \| \| --- \| \| 56 \| \| 14 \| \| 1 \| | \| **PD-L1 Range** \| \| --- \| \| PD-L1 Test ≥ 50% \| \| PD-L1 Test 1- 49% \| \| PD-L1Test Negative \| | \|  \| \| --- \| \| 78 \| \| 45 \| \| 64 \| | \|  \| \| --- \| \| 16 \| \| 12 \| \| 12 \| |
| \| **Race** \| \| --- \| \| White \| \| Asian \| \| Black \| \| Other \| | \|  \| \| --- \| \| 159 \| \| 59 \| \| 12 \| \| 53 \| | \|  \| \| --- \| \| 44 \| \| 10 \| \| 1 \| \| 15 \| | \| **irAE** \| \| --- \| \| irAE Occurred \| \| No irAE \| | \|  \| \| --- \| \| 122 \| \| 161 \| | \|  \| \| --- \| \| 30 \| \| 41 \| |
| \| **Stage** \| \| --- \| \| Stage I or II \| \| Stage III \| \| Stage IV \| | \|  \| \| --- \| \| 14 \| \| 29 \| \| 226 \| | \|  \| \| --- \| \| 2 \| \| 11 \| \| 57 \| | \| **irAE Timing** \| \| --- \| \| Early irAE \| \| Late irAE \| | \|  \| \| --- \| \| 59 \| \| 63 \| | \|  \| \| --- \| \| 17 \| \| 13 \| |
| \| **Smoking Status** \| \| --- \| \| Prior/Current Smoking \| \| Non-Smoking \| | \|  \| \| --- \| \| 191 \| \| 92 \| | \|  \| \| --- \| \| 51 \| \| 20 \| | \| **Type of irAE** \| \| --- \| \| Non-specific \| \| Categorized \| | \|  \| \| --- \| \| 60 \| \| 62 \| | \|  \| \| --- \| \| 16 \| \| 14 \| |

**Supplemental Table 2: Somatic mutation frequencies in the non-small cell lung cancer (NSCLC) patients receiving an immune checkpoint inhibitor (ICI) treated at the City of Hope.** All National Comprehensive Cancer Network (NCCN) screening genes along with other commonly mutated genes in NSCLC patients that were included in the molecular tests in the cohort were evaluated.

|  | **Patients on ICI with a TKI (n=63, %)** | **Patients on ICI without a TKI (n=291, %)** | **All patients on ICI**  **(N=354, %)** |
| --- | --- | --- | --- |
| **TP53** | 27 (42.86%) | 99 (34.02%) | 126 (35.80%) |
| **EGFR** | 43 (68.25%) | 5 (1.72%) | 48 (13.56%) |
| **KRAS** | 2 (3.17%) | 32 (11%) | 34 (9.66%) |
| **CDKN2A** | 2 (3.17%) | 32 (11%) | 34 (9.66%) |
| **STK11** | 3 (4.76%) | 23 (7.9%) | 26 (7.39%) |
| **PIK3CA** | 6 (9.52%) | 20 (6.87%) | 26 (7.39%) |
| **SMARCA4** | 1 (1.59%) | 16 (5.5%) | 17 (4.83%) |
| **ERBB2** | 2 (3.17%) | 13 (4.47%) | 15 (4.26%) |
| **CDKN2B** | 0 (0%) | 15 (5.15%) | 15 (4.26%) |
| **ARID1A** | 1 (1.59%) | 11 (3.78%) | 12 (3.41%) |
| **LRP1B** | 1 (1.59%) | 10 (3.44%) | 11 (3.13%) |
| **ALK** | 7 (11.11%) | 1 (0.34%) | 8 (2.26%) |
| **BRAF** | 4 (6.35%) | 1(0.34%) | 5 (1.42%) |
| **MET** | 2 (3.17%) | 3 (1.03%) | 5 (1.42%) |
| **RET** | 0 (0%) | 4 (1.37%) | 4 (1.14%) |
| **ROS1** | 0 (0%) | 1(0.34%) | 1 (0.28%) |
| **NTRK1** | 0 (0%) | 0 (0%) | 0 (0%) |
| **NTRK2** | 0 (0%) | 0 (0%) | 0 (0%) |
| **NTRK3** | 0 (0%) | 0 (0%) | 0 (0%) |

**Supplemental Table 3: Comparison of immune checkpoint inhibitor (ICI) therapy approach, tyrosine kinase inhibitor (TKI) targets, and overall survival (OS) time of patients receiving ICI prior to TKI therapy and those receiving ICI after TKI therapy.** Count data is reported for each prospective group with the % of the population reported in parentheses. Median overall survival time median and the interquartile range (IQR) is reported in the table.

|  | **ICI Prior to TKI therapy**  (n= 10) | **ICI Therapy After TKI Therapy** (n= 53) |
| --- | --- | --- |
| **Immunotherapy Combination Status**  ICI Monotherapy | 5 (50%) | 40 (75.5%) |
| ICI with Chemotherapy | 3 (30%) | 8 (15.1%) |
| Combination ICI Therapy | 2 (20%) | 5 (9.4%) |
| **TKI Therapy Targets**  Anti-EGFR TKI | 6 (60%) | 42 (79.2%) |
| Anti-ALK TKI | 2 (20%) | 8 (15.1%) |
| Anti-BRAF TKI | 1 (10%) | 2 (3.8%) |
| Anti-MET TKI | 1 (10%) | 0 (0%) |
| **Overall Survival Time**  Median (months)  Interquartile range (months) | 24.6  14.7- 27.6 | 7.57  3.2- 13.1 |

**Supplemental Table 4: Coefficients of baseline and optimized logistic regression models predicting 1-year overall survival (OS) and 6-month real-world progression free survival (rwPFS).**

Significant variables with P-values < 0.05 are noted with * symbols in the table; variables excluded in optimized logistic regression models are left blank in the table. Variables having p-values with 2 asterisks (**) have P-values < 0.01, and 3 asterisks (***) have p-values < 0.001.

|  | **Baseline logistic regression 1-Year OS** | **Baseline logistic regression 6-month rwPFS** | **Optimized logistic regression 1-Year OS** | **Optimized logistic regression 6-Month rwPFS** |
| --- | --- | --- | --- | --- |
| *Male* | 0.054 | 0.058 |  |  |
| *Age at Diagnosis* | -0.007 * | -0.006 * | -0.007 * | -0.006 * |
| *Adenocarcinoma* | -0.046 | 0.02 |  |  |
| *Squamous* | -0.076 | 0.009 |  |  |
| *White* | -0.012 | -0.10 |  |  |
| *Asian* | 0.031 | -0.09 |  |  |
| *Black* | 0.047 | -0.26 |  |  |
| *Smoking* | -0.083 | 0.019 |  |  |
| *Stage I or II* | 0.281 | 0.022 | 0.26 |  |
| *Stage III* | 0.036 | 0.08 |  |  |
| *Stage IV* | -0.138 | -0.156 | -0.156 * | -0.209 ** |
| *anti-pd-1* | -0.269 | 0.037 |  |  |
| *anti-pd-l1* | -0.424 | -0.062 | -0.163 * | -0.096 |
| *prior tyrosine kinase inhibitor (tki) therapy* | -0.257 ** | -0.262 ** | -0.221 ** | -0.290 *** |
| *general immune-related adverse events (irAEs)* | 0.165 | 0.157 | 0.168 | 0.166 |
| *categorized irAE* | 0.345 *** | 0.308 ** | 0.338 *** | 0.310 ** |
| *early irAE* | -0.239 * | -0.266 * | -0.241 * | -0.264 * |
| *PDL1 test negative* | -0.014 | -0.048 |  |  |
| *PDL1 range 1 to 49%* | -0.036 | 0.121 |  | 0.129 |
| *PDL1 range 50 to 100%* | 0.141 | 0.139 | 0.15 * | 0.168 * |

**Supplemental Figures**

**
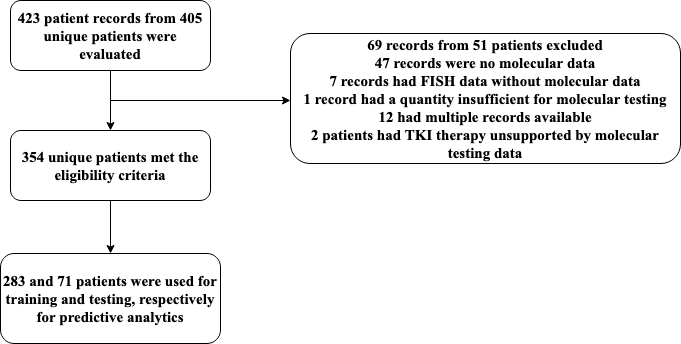
**

**Supplemental Figure 1:** Patient selection based on study eligibility criteria. 423 patient records were reviewed. 69 patient records from 51 unique patients were excluded. Finally, 354 patients out of 423 patient records remained for analyses.


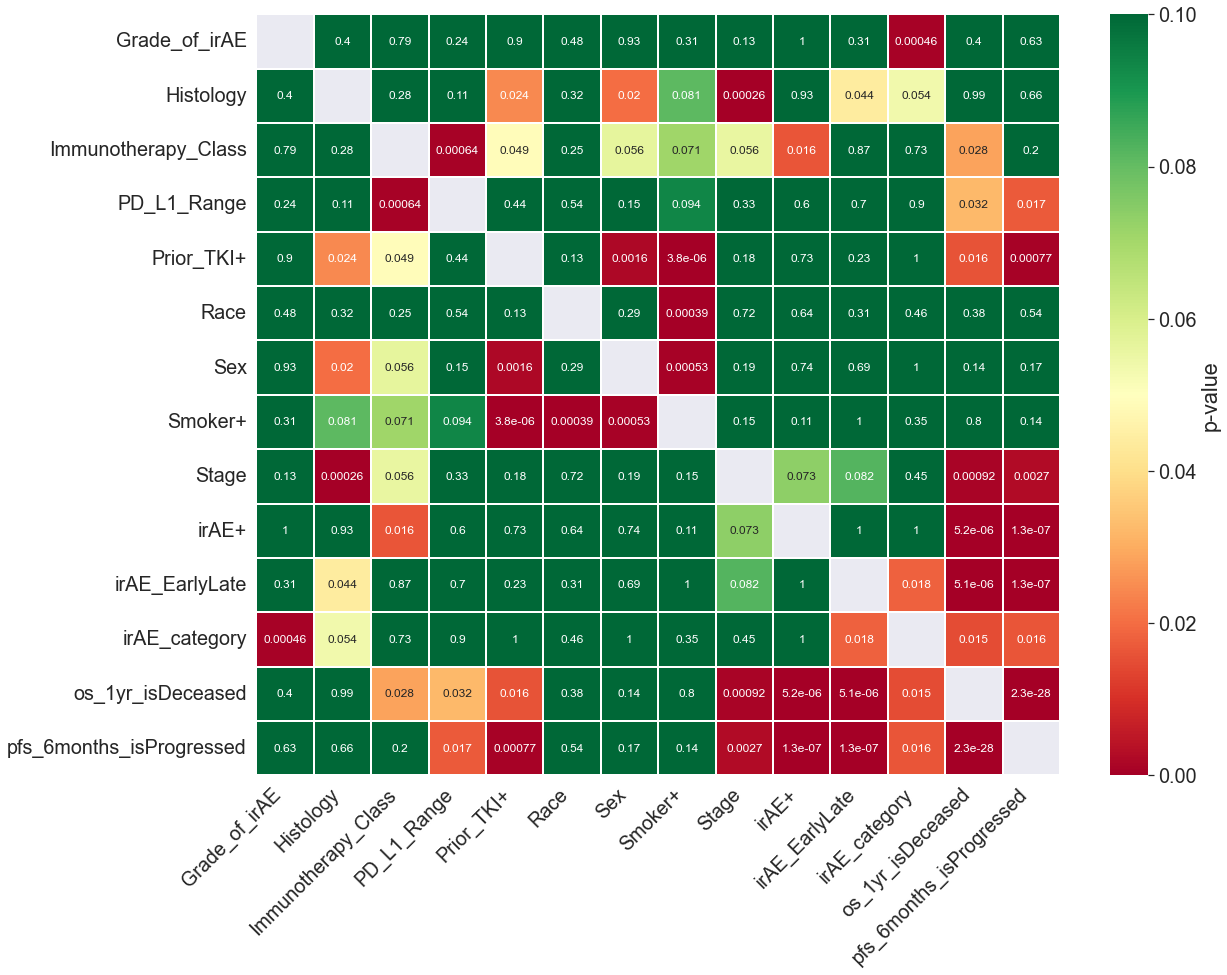


**Supplemental Figure 2: Heatmap showing the association between patient demographic features and survival outcome in the training data set.** Each box within the heatmap denotes a p-value of association between pairs of categorical variables derived from the Chi-squared test of independence. A smaller p-value is indicated darker red in the color map. irAE: immune-related adverse event; PD_L1: programmed death-ligand 1; Prior_TKI: tyrosine kinase inhibitor therapy prior to the immunotherapy; Stage: stage of cancer; irAE_EarlyLate: timing of onset of irAE (earlier or later than the median value).

**
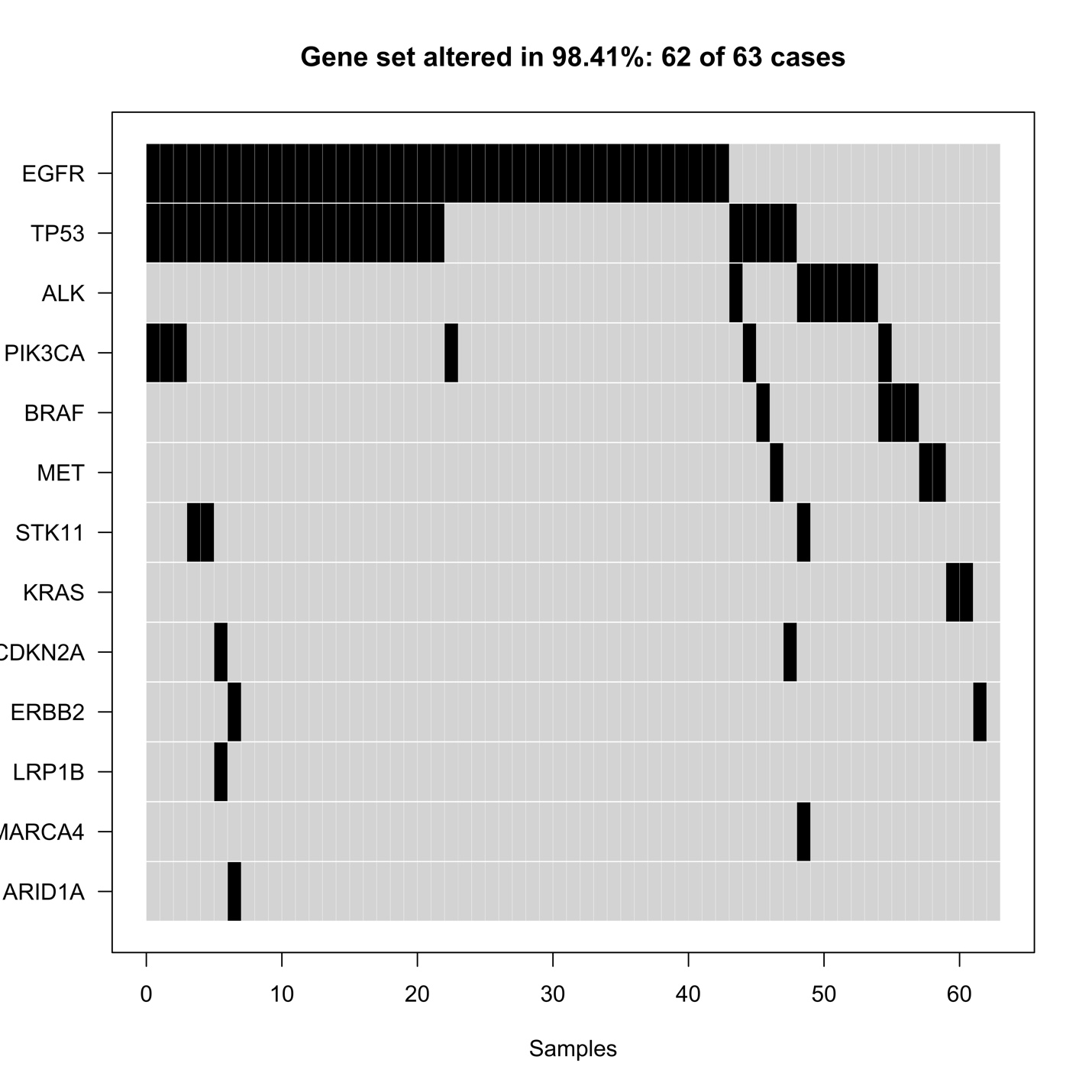
**

**Supplemental Figure 3:** Co-mutation plot of 63 non-small cell lung cancer (NSCLC) patients who previously had tyrosine kinase inhibitor therapies prior to receiving treatment with immune checkpoint inhibitors at the City of Hope. All patients had an actionable mutation. One patient’s genomic data was blinded due to participation in clinical trial. Frequent gene mutations along with genes indicated for tyrosine kinase inhibitors are shown. *EGFR, TP53, ALK and PI3KCA* were the top somatically mutated genes in this cohort. No somatic mutation in *RET, CDKN2B, NTRK1/2/3* has been found in this cohort.


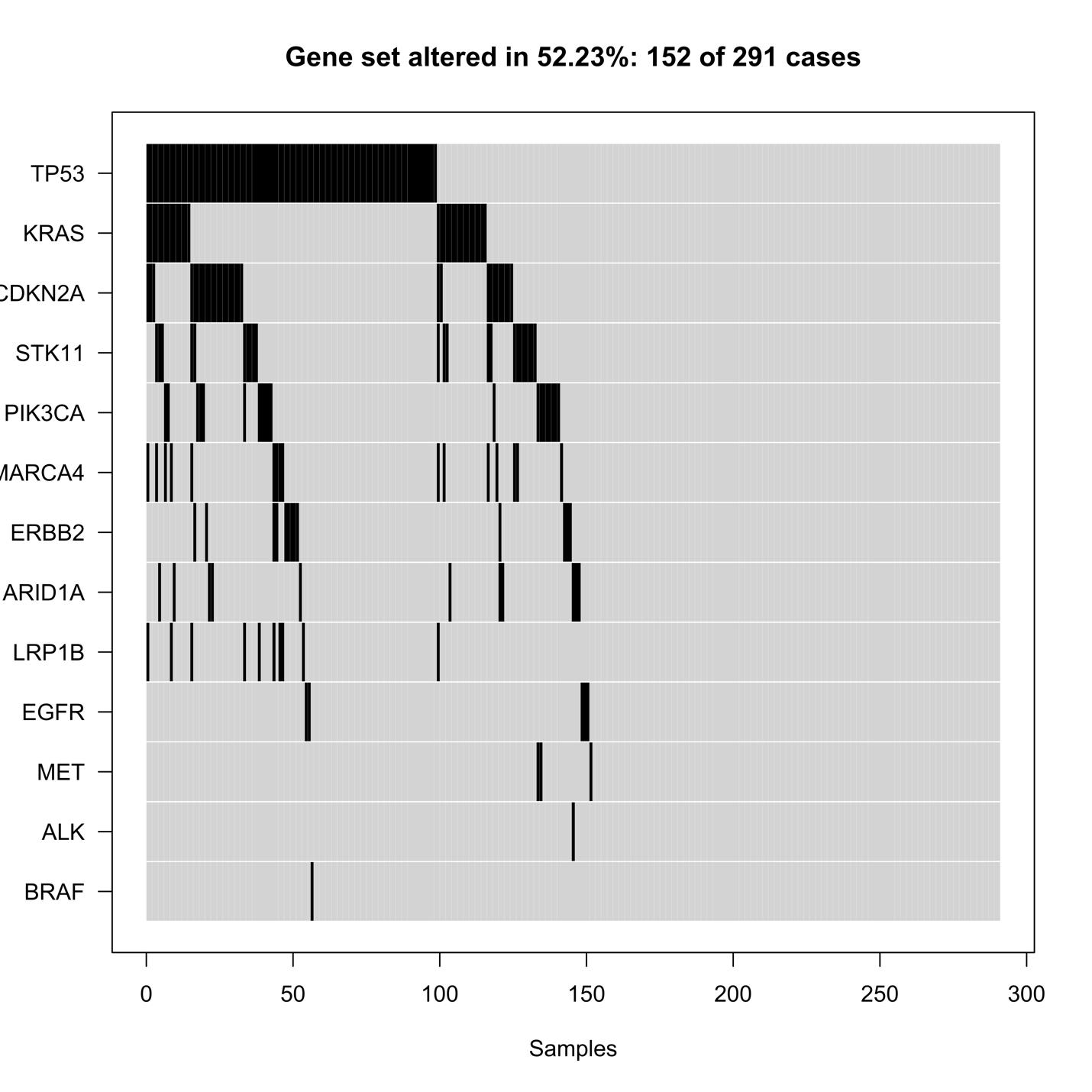


**Supplemental Figure 4**: Co-mutation plot of 291non-small cell lung cancer (NSCLC) patients without any prior tyrosine kinase inhibitor therapies treated with immune checkpoint inhibitors at the City of Hope. Frequent gene mutations along with genes indicated for tyrosine kinase inhibitors are shown. *TP53, KRAS* and *CDKN2A* were the top somatically mutated genes in this cohort. No somatic mutation in *RET*, *NTRK1/2/3* has been found in this cohort.

**Supplemental Figure 5: Kaplan Meier survival curve comparing survival of patients receiving tyrosine kinase inhibitor (TKI) therapy prior to immune checkpoint inhibitor (ICI) therapy to patients receiving TKI therapy after ICI therapy. (A)** and **(B)** compare patients for overall survival (OS) and real-world progression free survival (rwPFS), respectively. P-values less than 0.05 indicates a statistically significant probability from the log-rank statistical test.


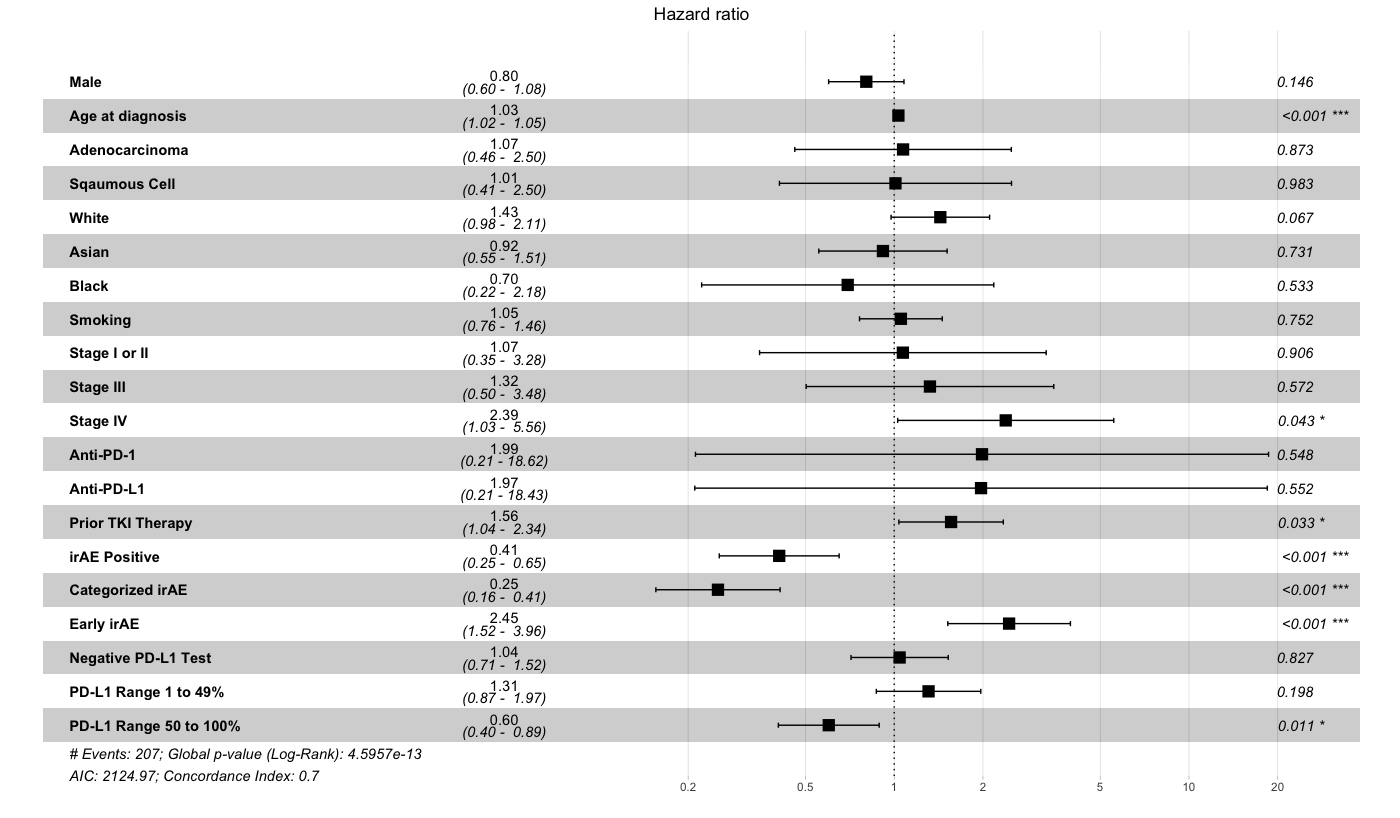


**Supplemental Figure 6: Forest plot of hazard ratios for multivariate Cox-proportional hazards model for overall survival.**

From left to right, the variable is listed followed by its hazard ratio and 95% confidence interval in parenthesis below it. The graphic representation has square points representing the hazard ratio value and bracketed lines representing its 95% confidence interval. P-values are shown on the right with significant variables. * P-value < 0.05; ** P-values < 0.01; ***P-values < 0.001.


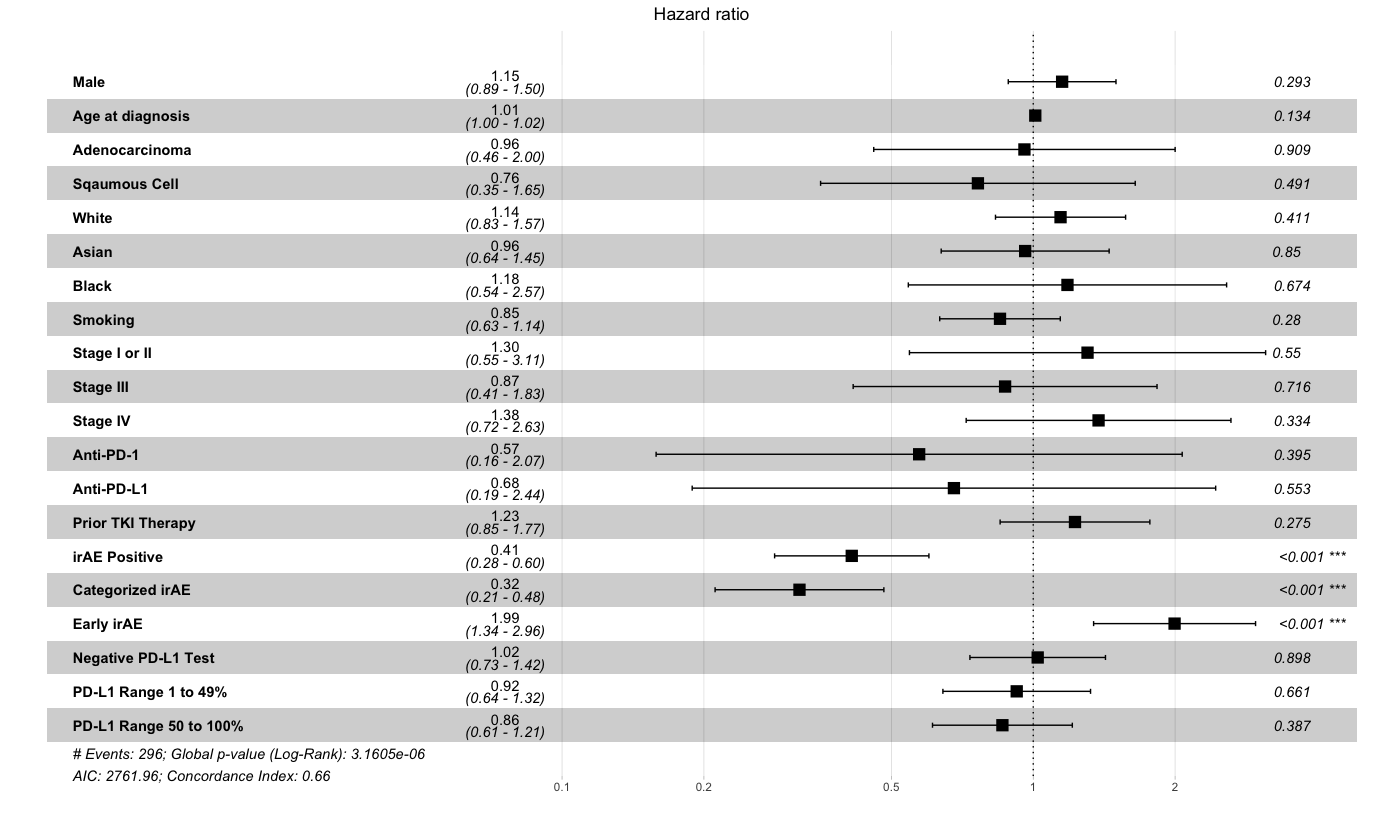


**Supplemental Figure 7: Forest plot of hazard ratios for multivariate Cox-proportional hazards model for real-world progression-free survival (rwPFS).** From left to right, the variable is listed followed by its hazard ratio and 95% confidence interval in parenthesis below it. The graphic representation has square points representing the hazard ratio value and bracketed lines representing its 95% confidence interval. P-values are shown on the right with significant variables. * P-value < 0.05; ** P-values < 0.01; ***P-values < 0.001.

**
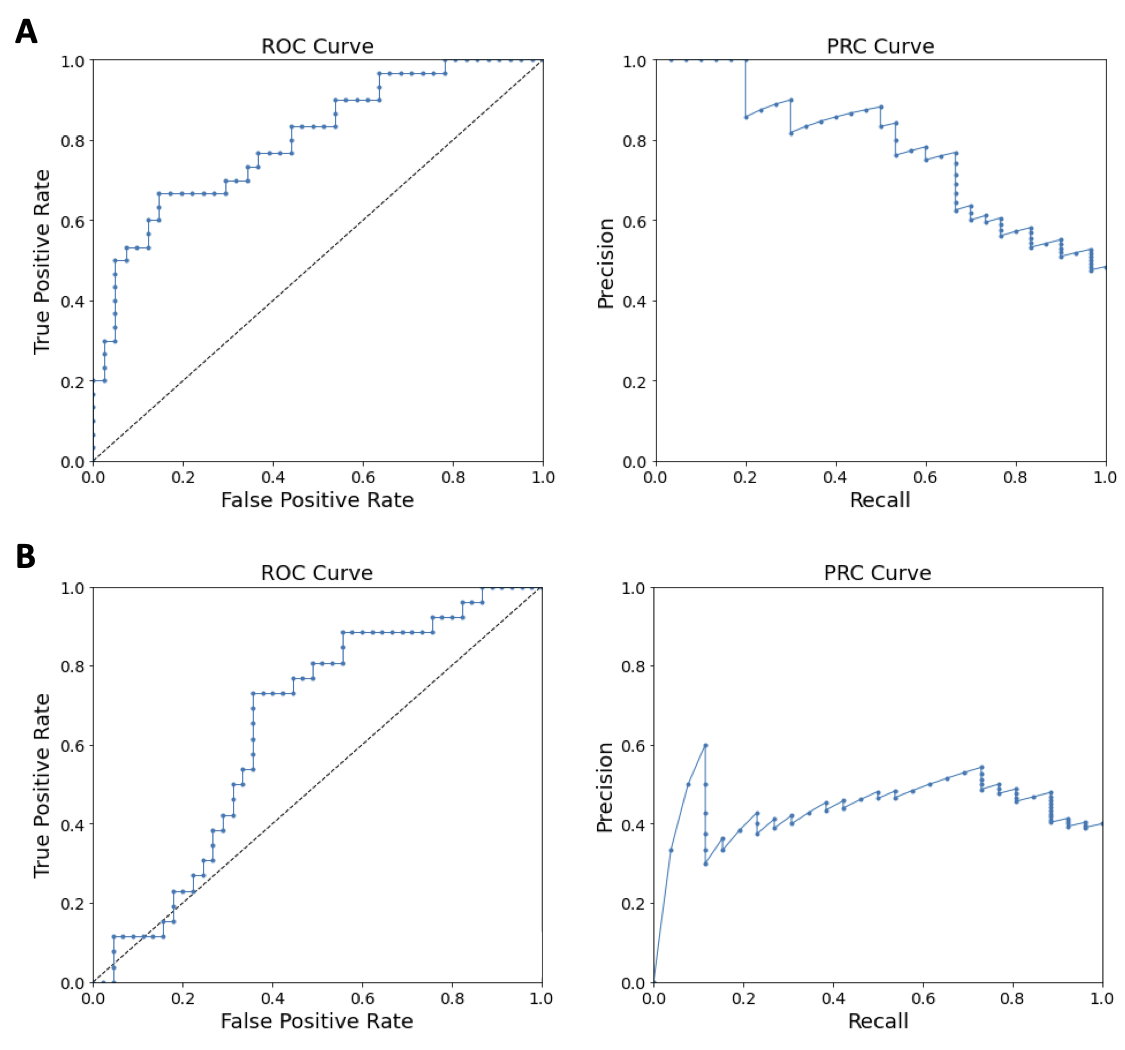
**

**Supplemental Figure 8: Evaluation of prediction performance of elastic-net logistic regression on the holdout test data set by using receiver operating characteristic (ROC) curve plot and precision-recall (PRC) curve plot for (A) overall survival (OS) model and (B) real world progression-free survival (PFS) model.** For the OS model, the area under the ROC curve (AUROC) was 0.80 and the area under the PRC curve (AUPRC) was 0.74. For the PFS model, the AUROC was 0.65 and the AUPRC was 0.44.
